# Is there any difference in urinary continence between bilateral and unilateral nerve sparing during radical prostatectomy? A systematic review and meta-analysis

**DOI:** 10.1186/s12957-024-03340-6

**Published:** 2024-02-23

**Authors:** Peng Xiang, Zhen Du, Di Guan, Wei Yan, Mingdong Wang, Danyang Guo, Dan Liu, Yuexin Liu, Hao Ping

**Affiliations:** Department of Urology, Beijing Tongren Hospital, Capital Medical University, No1. Dongjiaominxiang Street, Dongcheng District, Beijing, 100730 China

**Keywords:** Radical prostatectomy, Urinary continence, Bilateral nerve sparing, Unilateral nerve sparing, Systematic review

## Abstract

**Context:**

In men with prostate cancer, urinary incontinence is one of the most common long-term side effects of radical prostatectomy (RP). The recovery of urinary continence in patients is positively influenced by preserving the integrity of the neurovascular bundles (NVBs). However, it is still unclear if bilateral nerve sparing (BNS) is superior to unilateral nerve sparing (UNS) in terms of post-RP urinary continence. The aim of this study is to systematically compare the differences in post-RP urinary continence outcomes between BNS and UNS.

**Methods:**

The electronic databases of PubMed and Web of Science were comprehensively searched. The search period was up to May 31, 2023. English language articles comparing urinary continence outcomes of patients undergoing BNS and UNS radical prostatectomy were included. Meta-analyses were performed to calculate pooled relative risk (RR) estimates with 95% confidence intervals for urinary continence in BNS and UNS groups at selected follow-up intervals using a random-effects model. Sensitivity analyses were performed in prospective studies and robotic-assisted RP studies.

**Results:**

A meta-analysis was conducted using data from 26,961 participants in fifty-seven studies. A meta-analysis demonstrated that BNS improved the urinary continence rate compared to UNS at all selected follow-up points. RRs were 1.36 (1.14–1.63; *p* = 0.0007) at ≤ 1.5 months (mo), 1.28 (1.08–1.51; *p* = 0.005) at 3–4 mo, 1.12 (1.03–1.22; *p* = 0.01) at 6 mo, 1.08 (1.05–1.12; *p* < 0.00001) at 12 mo, and 1.07 (1.00-1.13; *p* = 0.03) at ≥ 24 mo, respectively. With the extension of the follow-up time, RRs decreased from 1.36 to 1.07, showing a gradual downward trend. Pooled estimates were largely heterogeneous. Similar findings were obtained through sensitivity analyses of prospective studies and robotic-assisted RP studies.

**Conclusion:**

The findings of this meta-analysis demonstrate that BNS yields superior outcomes in terms of urinary continence compared to UNS, with these advantages being sustained for a minimum duration of 24 months. It may be due to the real effect of saving the nerves involved. Future high-quality studies are needed to confirm these findings.

**Supplementary Information:**

The online version contains supplementary material available at 10.1186/s12957-024-03340-6.

## Introduction

Globally, prostate cancer (PCa) is the second most common cancer and the fifth leading cause of cancer death among men in 2020, with approximately 1,414,259 new cases and 375,000 deaths [1, 2]. Radical prostatectomy (RP) is one of the gold standard treatments for patients diagnosed with localized or locally advanced prostate cancer. It is widely acknowledged that urinary incontinence and erectile dysfunction are significant causes of low quality of life in many surgical men [2, 3]. At 12 months (mo), 16% of post-RP men are incontinent (using a no-pad definition) [4]. The reported potency rates following robot-assisted RP are highly variable, ranging from 54–90% [5]. Long-term urinary incontinence has been linked to a number of complex issues in individuals with prostate cancer, such as obsessive-compulsive about restroom locations and preventing leaks, as well as feelings of shame, helplessness, and uncleanliness when control is compromised [6].

In RP, different surgical approaches (open, laparoscopic, or robotic-assisted) are used, but postoperative continence is mainly determined by surgical technique, including preservation techniques and reconstructing techniques [2, 7]. In general, the retention of various structures like the neurovascular bundles (NVBs) and bladder neck aids in the control of urination [8]. The preservation of NVBs during RP has been shown to lead to an earlier return of continence [6, 9]. Recent meta-analyses reported that patients who had any nerve sparing (NS) surgery (i.e., bilateral nerve sparing, unilateral nerve sparing, or unspecified) had significantly better continence outcomes compared to those who had non-nerve sparing surgery [3, 6, 9]. Furthermore, Reeves et al. [6] demonstrated that there was only a statistically significant difference in continence outcomes between bilateral nerve sparing (BNS) and unilateral nerve sparing (UNS) at short-term follow-up (≤ 1.5 mo). However, Nguyen et al. [3] indicated that lower rates of urinary incontinence were significantly observed with BNS compared to UNS at 1 year. If sparing two-sided NVBs has a real advantage in postoperative urinary continence, then preservation of continence should be an independent indication for BNS, which will be crucial for clinical practice. Until now, data concerning bilateral vs. unilateral nerve-sparing radical prostatectomy in urinary continence has been widely reported and the results are controversial.

In this research, we aimed to conduct a systematic review and meta-analysis to assess whether there were differences in urinary continence outcomes after RP surgery between BNS and UNS in both short-term and long-term follow-up. The hypothesis posits that adopting a BNS approach may mitigate the incidence of urinary incontinence.

## Methods

A systematic review and meta-analysis was conducted in accordance with the Preferred Reporting Items for Systematic Reviews and Meta-analysis (PRISMA) statement. The protocol for this study was registered with PROSPERO (CRD42022378340, https://www.crd.york.ac.uk/PROSPERO/#recordDetails).

### Search strategy and eligibility criteria

To identify potentially relevant studies, searches were conducted in the PubMed and Web of Science electronic databases on May 31, 2023. Based on the absence of a formal description of NS surgery before 1982, the search was limited to studies published after that time.

We included studies reporting the outcomes of urinary continence in men treated for PCa with a BNS RP (intervention) and a UNS RP (control). Specifically, this review included retrospective and prospective studies that evaluated the comparison of urinary incontinence outcomes between UNS and BNS. Cross-sectional studies and observational studies without a control group (i.e., single-cohort studies) were not included. The present study did not attempt to analyze more specific or alternative types of nerve sparing, such as intra/interfascial versus standard, risk-stratified NS, or sural nerve grafting. We did not exclude studies based on surgical approaches such as open RP (ORP), laparoscopic RP (LRP), or robotic-assisted RP (RARP). In the search, the following terms were used: [“nerve sparing”] AND [“prostatectomy”] AND [“unilateral “OR “bilateral”]. The above keywords are searched using “all fields”. See Additional files 1 and  2 for detailed search strategies.

A meta-analysis of relevant prospective and retrospective studies with sufficient data was conducted. The scope of the review according to the PICO process (Patient, Intervention, Comparison, Outcomes) is as follows: P-patients with prostate cancer; I- radical prostatectomy with BNS; C-radical prostatectomy with UNS; O-urinary continence outcomes. The search was limited to English-language publications. To identify additional potentially relevant studies, we manually searched the reference lists of relevant publications and reviews. When data is duplicated, more recent or comprehensive studies are preferred.

### Outcome

Postoperative urinary continence was the primary outcome of this systematic review and meta-analysis. Continence rates from included studies were pooled in this meta-analysis. We investigate the effect of BNS versus UNS on continence rates at selected follow-up intervals including ≤ 1.5 mo, 3–4 mo, 6 mo, 12 mo, and ≥ 24 mo.

### Study selection and data extraction

Two authors screened the search results (titles and abstracts), and any disagreements were resolved by consensus. In line with the previously outlined selection criteria, the full texts of all potentially relevant publications were retrieved for review.

Data extracted involved patient age, sample size, continence definition, and surgical approach. To conduct the meta-analysis, the total number of participants and events were extracted (defined as a number of continent men). Raw numbers can also be calculated based on hazard ratios, relative risks, or odds ratios results. Any differences of opinion regarding data extraction were resolved through discussion and, if necessary, consultation with a third author to reach an agreement.

### Synthesis of results and statistical analysis

The risk of bias in each study was independently evaluated by two authors according to the method published in the study of Reeves et al. [6]. This evaluation covered baseline continence, outcome assessment (the definition of urinary continence in each paper), comparability of groups (comparison of clinical information including age, tumor stage, and Gleason classification), NS assessment (details of NS surgical procedures), surgical technique variations (additional surgical procedures, such as puboprostatic ligaments preservation, bladder neck reconstruction and posterior rhabdosphincter reconstruction) and other issues like unexplained loss to follow-up and selective outcome reporting. Each comparison was measured as a risk ratio (RR) with 95% confidence interval. Recovery of continence is more likely in the intervention group if the RR > 1.0.

Results of each study were grouped by type of NS (BNS, or UNS), as well as stratified by the timing of outcome reporting. Based on all available results in included studies, the outcome timing categories are ≤ 1.5 mo, 3–4 mo, 6 mo, 12 mo, and ≥ 24 mo. A Mantel-Haenszel random-effects model was used to calculate pooled.

RRs for each time category. The I^2^ statistic (I^2^ ≥ 50%) and chi-square test (*p* ≤ 0.10) were used to assess heterogeneity.

Sensitivity analysis was conducted for studies that used a prospective design or prospectively collected data. At the same time, a sensitivity analysis of RARP studies was also conducted. Moreover, due to few studies, it is unlikely that separate sub-analyses could be performed based on the type of procedure (e.g., robotic-assisted RP versus other RP) and urinary continence definition (e.g., no pad vs. other).

To assess the risk of publication bias, funnel plots and the Egger test of funnel plot symmetry were generated for all primary outcomes. The Egger test is based on linear regression of the intervention effect estimate against its standard error weighted by the inverse of the intervention effect estimate’s variance. Examine potential publication bias through visual examination of funnel plots. Importantly, the significant publication bias is indicated by a p-value < 0.05. RevMan5.3 and STATA 12 software were used to perform all statistical analyses and generate forest plots.

## Results

### Study selection and characteristics

Our search yielded 1249 unique records. Ultimately, fifty-seven studies were included for quantitative synthesis. A total of forty-six prospective longitudinal cohort studies [10–55] and eleven retrospective cohort studies [56–66] were included in this review (Table 1). The study sample size ranged from 15 to 2019 participants, with a total of 26,961 patients included in the final analysis. A series of studies were conducted between 1997 and 2023. Most patients included in the studies were undergoing RARP. Some studies used open surgery (retropubic or perineal) or extraperitoneal laparoscopic surgery. About 90% of the research originated from Europe and the United States. The demographic data of fifty-seven patient-series were comparable, with an average age range of 51–69 years and an average follow-up range of immediately after catheter removal to > 60 mo. The most common definition of urinary continence in included studies was no pad use.

**Table 1:** Characteristics of the included studies

Study, year	Sample size	Surgical approach	Age, yr, mean or median	Timing of outcome, mo	Continence definition
**Prospective**					
Albayrak 2010 [10]	BNS 73, UNS 12	Perineal	62	3	No pad
Asimakopoulos 2019 [11]	BNS 69, UNS 10	RARP	65	Immediate	No pad
Avulova 2018 [12]	BNS 805, UNS 186	80% RARP, 19% ORP, 1% other	BNS 61, UNS 63	36	No pad
Berg 2014 [13]	BNS 85, UNS 72	RARP	BNS 60, UNS 63	3, 6, 12, 24	No pad
Berry 2009 [14]	BNS 341, UNS 89	65%RARP, 12% LRP, 23% ORP	BNS 59, UNS 59	36	Return to 75% of baseline continence score (UCLA-PCI)
Bhat 2022 [15]	BNS 1308, UNS 532	RARP	51	12	No pad
Budaus 2009 [16]	BNS 464, UNS 173	Retropubic	63	12	0–1 pad per day
Burkhard 2006 [17]	BNS 75, UNS 322	Retropubic	64	12	No pad
Choi 2011 [18]	BNS 469, UNS 89	RARP	BNS 58, UNS 59	4, 12, 24	No pad
Collette 2021 [19]	BNS 990, UNS 466	RARP	66	12	0–1 pad per day
d’Altilia 2022 [20]	BNS 120, UNS 49	59%RARP; 41% ORP	66	3, 6, 12	24-h pad test ≤ 20g/day
Dalkin 2006 [21]	BNS 53, UNS 68	Retropubic	63	12, 24	No pad
El-Hakim 2015 [22]	BNS 167, UNS 28	RARP	60	3	No pad
Feng 2020 [23]	BNS 187, UNS 84	RARP	62	15	No pad
Fossati 2017 [24]	BNS 1351, UNS 144	RARP	63	12	No pad
Geraerts 2013 [25]	BNS 112, UNS 44	36%RARP, 64%ORP	62	12	0 g urine leakage
Hatiboglu 2015 [26]	BNS 697, UNS 202	57%RARP, 43%Retropubic	64	Immediate	No pad
Hinata 2014 [27]	BNS 35, UNS 92	RARP	BNS 62, UNS 63	1, 3, 6	No pad
Holze 2019 [28]	BNS 153, UNS 57	46.2%RARP, 53.8%ORP	65	3	No pad
Kim 2019 [29]	BNS 285, UNS 214	RARP	65	1, 3, 6, 12	No pad and no leakage
Ko 2012 [30]	BNS 779, UNS 394	RARP	60	1.5, 3, 6	No pad and no leakage
Kohjimoto 2022 [31]	BNS 92, UNS 199	RARP	69	1, 3, 6, 12, 24	No pad
Kováčik 2019 [32]	BNS 60, UNS 28	RARP	64	0.5, 3, 6, 12	0–1 pad per day
Kowalczyk 2013 [33]	BNS 490, UNS 120	RARP	60	5, 12	No pad
Kung 2015 [34]	BNS 33, UNS 10	Retropubic	60	57	No pad
Lavigueur-Blouin 2015 [35]	BNS 201, UNS 52	RARP	60	1	No pad
Lee 2010 [36]	BNS 58, UNS 15	RARP	59	1.5	No pad
Marien 2008 [37]	BNS 538, UNS 72	Retropubic	BNS 57, UNS 59	24	Total control or occasional leakage
Nandipati 2007 [38]	BNS 66, UNS 25	ORP	BNS 62, UNS 64	3, 6, 12, 24, > 60	No pad
Novara 2010 [39]	BNS 201, UNS 22	RARP	62	12	No leakage
Nyarangi-Dix 2020 [40]	BNS 156, UNS 86	RARP	65	12, 24	No pad
Pagliarulo 2020 [41]	BNS 163, UNS 68	LRP	65	12	No pad
Pick 2011 [42]	BNS 357, UNS 143	RARP	BNS 60, UNS 63	1, 3, 12	No pad
Reichert 2022 [43]	BNS 25, UNS 27	RARP	64	12	No pad
Rigatti 2012 [44]	BNS 24, UNS 9	RARP	66	1, 3	No leakage
Sammon 2013 [45]	BNS 1015, UNS 125	RARP	60	Immediate	No pad
Scarcia 2018 [46]	BNS 208, UNS 201	RARP	65	1, 3, 12	0–1 pad per day
Steineck 2015 [47]	BNS 970, UNS 959	78%RARP, 22%ORP	63	12	No pad
Suardi 2012 [48]	BNS 900, UNS 49	Retropubic	64	12, 24	No pad
Talcott 1997 [49]	BNS 28, UNS 38	ORP	BNS 61, UNS 62	3, 12	No pad
Theissen 2019 [50]	BNS 76, UNS 23	50%RARP, 50%ORP	66	Immediate	Urine loss (≤ 10g) within 1h
Toren 2009 [51]	BNS 159, UNS 32	ORP	BNS 59, UNS 60	12	No or rare urine leakage
Tsikis 2017 [52]	BNS 396, UNS 51	RARP	58	12	No pad
Tzou 2009 [53]	BNS 73, UNS 112	Retropubic	63	12, 24	No pad
Van der Poel 2009 [54]	BNS 61, UNS 72	RARP	60	6	No involuntary urine loss
Van der Slot 2023 [55]	BNS 340, UNS 202	RARP	68	6, 12, 24	0–1 pad per day
**Retrospective**					
Chung 2020 [56]	BNS 125, UNS 72	70%RARP, 30%ORP	68	12	No pad
Fosså 2019 [57]	BNS 165, UNS 242	RARP	62	24	No pad
Greco 2011 [58]	BNS 250, UNS 207	Extraperitoneal LRP	BNS 59, UNS 60	1, 3, 12	No pad
Hinata 2019 [59]	BNS 46, UNS 137	RARP	BNS 65, UNS 66	24	No pad
Kadono 2015 [60]	BNS 15, UNS 36	RARP	BNS 64, UNS 65	12	24-h pad test ≤ 2g/day
Lee 2013 [61]	BNS 100, UNS 54	Extraperitoneal LRP	66	3	No pad
Noël 2022 [62]	BNS 391, UNS 138	RARP	57	1.5	No leakage
Palisaar 2015 [63]	BNS 1332, UNS 687	RARP or ORP	64	1.5	0–1 pad per day
Punnen 2014 [64]	BNS 157, UNS 74	RARP	NA	6	No pad
Shikanov 2011 [65]	BNS 1021, UNS 322	RARP	60	12	No pad
Wang 2014 [66]	BNS 2, UNS 13	RARP	64	12	No pad

*BNS* bilateral nerve sparing, *UNS* unilateral nerve sparing, *RARP* robot-assisted radical prostatectomy, *LRP *laparoscopic radical prostatectomy, *NA* not available, *ORP* open radical prostatectomy, *UCLA-PCI* University of California, Los Angeles, Prostate Cancer Index

### Assessment of methodology of included studies

Because the BNS/UNS group stratification characteristics were not fully described in most of the included studies, the baseline imbalance cannot be determined. In the case of the data provided, selection bias was evident. Patients who underwent the BNS procedure were more likely to have younger ages, lower Gleason scores, better baseline sexual function, and a favorable clinical stage. A few studies also adjusted for age, comorbidities, and history of lower abdominal surgery when calculating RRs. Indeed, urinary continence outcomes may be influenced by various confounding factors, including age, body mass index, prior surgical history, prostate volume, membranous urethral length, tumor staging and grading, and surgeon experience. However, despite the lack of thorough characterization of NVB preservation, even in patients with low-risk prostate cancer, its implementation was associated with a higher risk of cancer at the margins of the excised tissue [67].

Baseline continence was seldom reported. In several studies, there was no urinary incontinence reported before surgery. Several other studies have reported similar baseline continence scores in the comparison groups.

Few studies have described how to define or record the NS status. In one study, it is stated that > 70% of the bundles preserved in situ can be regarded as NS [18]. Several studies describe retrospective reviews of surgical reports to determine NS status. Some studies directly describe NS using interfascial, extrafascial, or intrafascial techniques. At the same time, some of the studies were conducted concurrently with the preservation of the puboprostatic ligament or bladder neck reconstruction.

The assessment of results was variable. Most studies used the definition of no-pad for continence. Few studies used validated instruments to determine incontinence status. Even when a validated tool was used, it was often unclear who submitted the questionnaire and whether the assessor was blinded. Potential selective outcome reporting or unexplained loss to follow-up was apparent in many studies (Additional file 3).

### Urinary continence outcomes

**Fig. 1:**
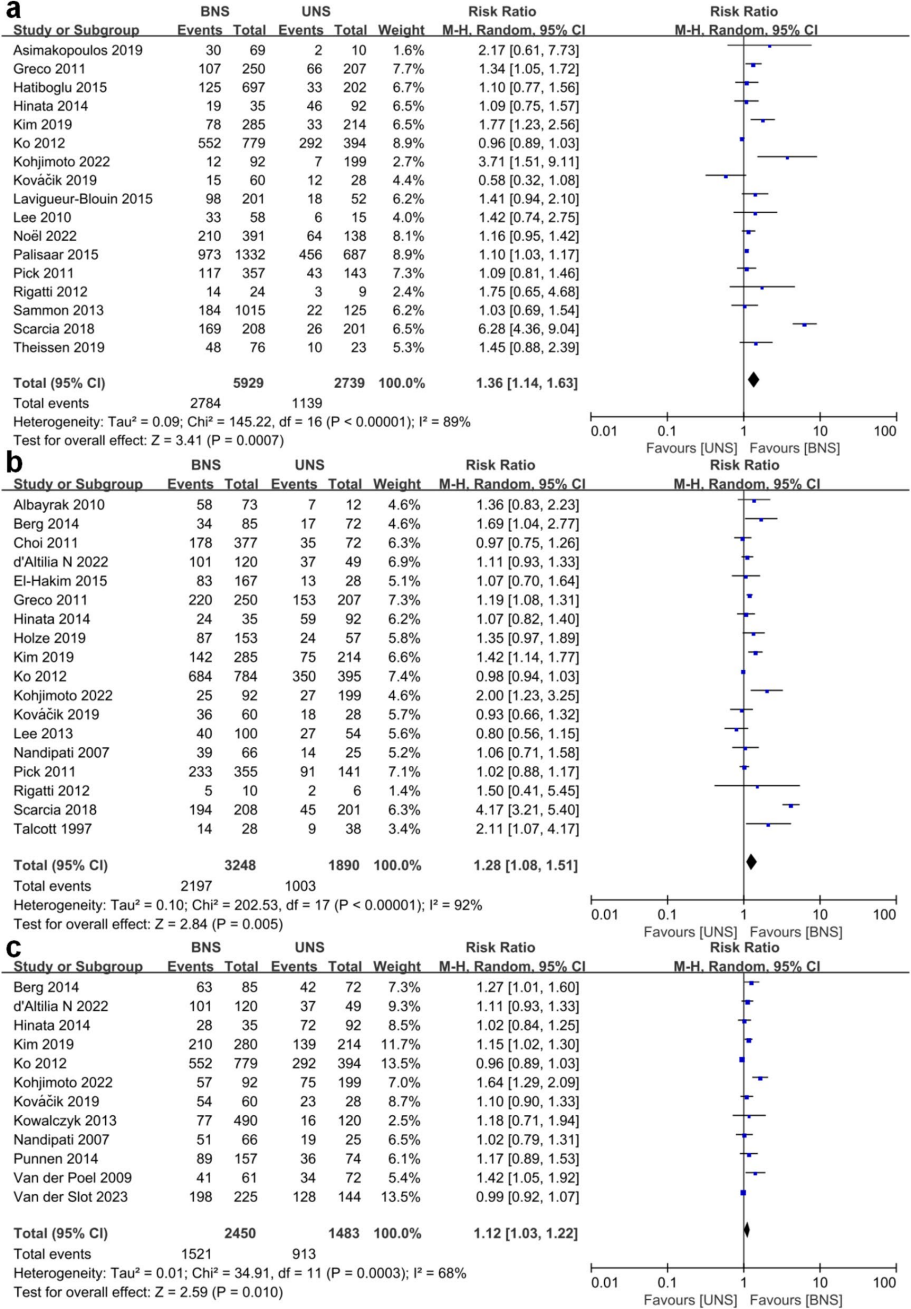
**a **Forest plot of continence rates for bilateral nerve sparing (BNS) versus unilateral nerve sparing (UNS) at ≤ 1.5 mo. **b **Forest plot of continence rates for bilateral nerve sparing (BNS) versus unilateral nerve sparing (UNS) at 3–4 mo. **c **Forest plot of continence rates for bilateral nerve sparing (BNS) versus unilateral nerve sparing (UNS) at 6 mo

**Fig. 2:**
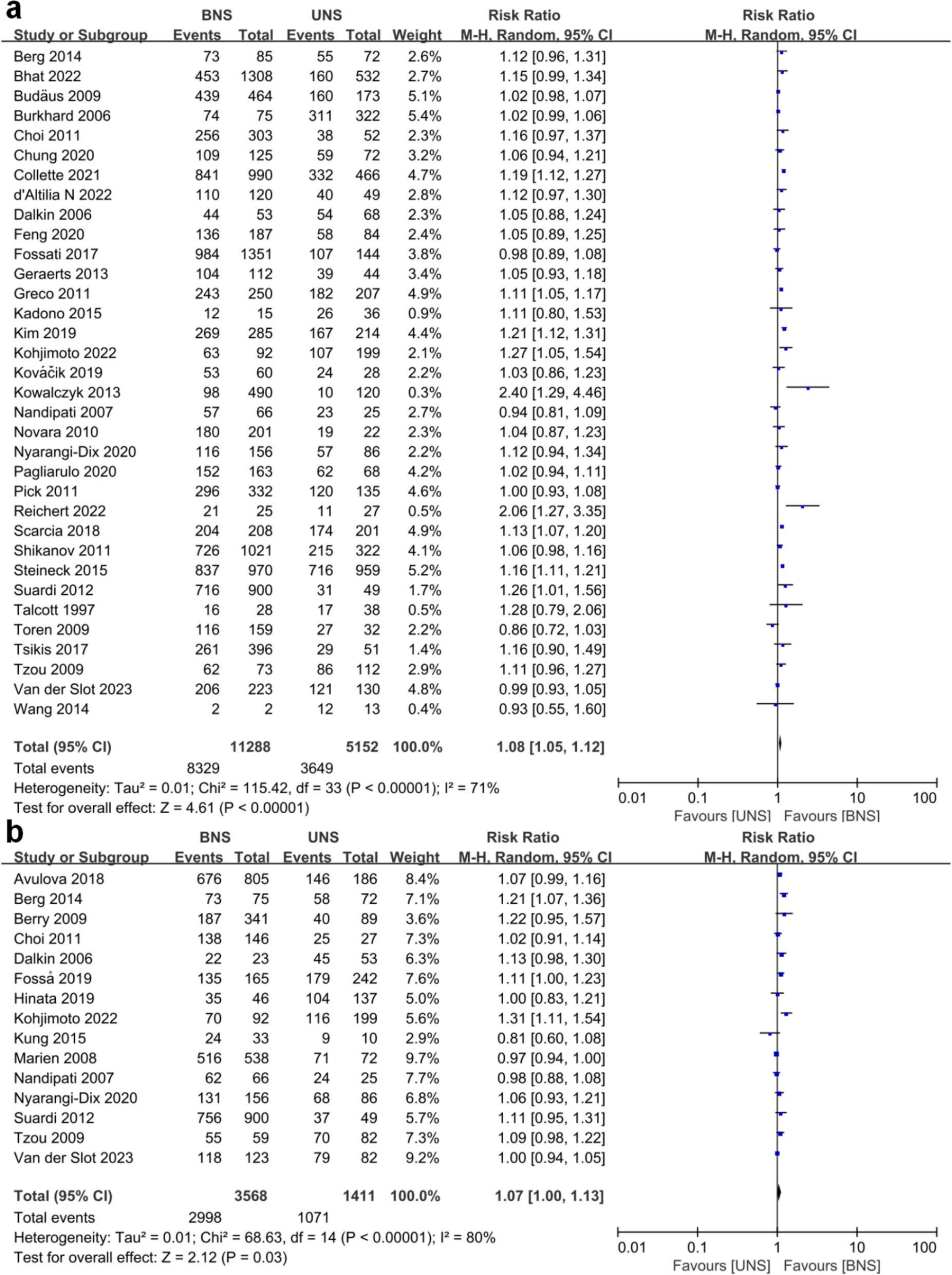
**a **Forest plot of continence rates for bilateral nerve sparing (BNS) versus unilateral nerve sparing (UNS) at 12 mo. **b **Forest plot of continence rates for bilateral nerve sparing (BNS) versus unilateral nerve sparing (UNS) at ≥ 24 mo

Patients who underwent the BNS procedure had significantly better continence outcomes compared to those who underwent the UNS procedure. RRs were 1.36 (1.14–1.63; *p* = 0.0007) at ≤ 1.5 mo, 1.28 (1.08–1.51; *p* = 0.005) at 3–4 mo, 1.12 (1.03–1.22; *p* = 0.01) at 6 mo, 1.08 (1.05–1.12; *p* < 0.00001) at 12 mo and 1.07 (1.00-1.13; *p* = 0.03) at ≥ 24 mo. Notably, the RRs decreased gradually as the follow-up time extended. Figures 1 and 2 showed the meta-analysis of urinary continence outcomes for BUS compared to UNS at selected follow-up points.

Sensitivity analysis revealed consistent overall results when prospective studies or RARP studies were considered alone. The RRs of BNS compared to UNS in prospective studies at ≤ 1.5 mo, 3–4 mo, 6 mo, 12 mo and ≥ 24 mo were 1.47 (1.05–2.04; *p* = 0.02), 1.33 (1.07–1.66; *p* = 0.01), 1.12 (1.02–1.23; *p* = 0.01), 1.09 (1.04–1.13; *p* < 0.0001) and 1.07 (1.00-1.14; *p* = 0.06), respectively (Additional files 4 and 5). The RRs of BNS compared to UNS in RARP patients at ≤ 1.5 mo, 3–4 mo, 6 mo, 12 mo and ≥ 24 mo were 1.47 (1.06–2.03; *p* = 0.02), 1.35 (1.00-1.81; *p* = 0.05), 1.14 (1.03–1.26; *p* = 0.01), 1.11 (1.05–1.16; *p* < 0.0001) and 1.09 (1.00-1.18; *p* = 0.04), respectively (Additional files 6 and 7).

### Heterogeneity and publication bias

Clinical and methodological inconsistencies were apparent in all included studies. There were significant differences in participant characteristics, including baseline urinary continence and age. Variations in surgical technique, such as preserving of the bladder neck, sparing of puboprostatic ligament, and posterior reconstruction, also varied from study to study or were not described in detail. Furthermore, some studies restricted surgery to high-volume surgeons or surgeons with a minimum level of expertise. In addition, there were also differences in the methods used to assess postoperative incontinence status. In general, high inter-study heterogeneity was observed at each time point of urinary continence. Consequently, a random-effects model was utilized in the meta-analyses. Statistical analysis of funnel plot asymmetry using Egger linear regression revealed no convincing evidence of publication bias, except for the outcome at 6 mo and ≥ 24 mo. Outcomes at 6 mo and ≥ 24 mo showed publication bias, possibly due to a limited number of included studies. P values for publication bias were 0.06, 0.06, 0.01, 0.21, and 0.01 at ≤ 1.5mo, 3–4 mo, 6mo, 12mo, and ≥ 24 mo, respectively (Additional file 8).

## Discussion

In this systematic review, we analyzed the differences in urinary continence between preserving bilateral NVBs and unilateral NVBs. The meta-analysis showed a correlation between BNS and improved urinary continence outcomes at all follow-up intervals, although this improvement gradually diminished with longer follow-up periods. Unlike the findings reported by Reeves et al. [6] and Nguyen et al. [3], which stated that BNS only exhibited good urinary continence at 1.5 months and 1 year after surgery, respectively.

The pathophysiology of post-RP urinary incontinence is not fully understood. Several factors are associated with the risk of postoperative urinary incontinence, including patient characteristics (e.g., body mass index, age, prostate volume, comorbidities) and provider-related factors (surgeon experience, skill, central volume, etc.) [68–70]. As far as the surgical methods are concerned, urethral sphincter preservation and nerve-sparing as well as the newly invented hood technique, can be effective in preventing post-RP urinary incontinence [70, 71]. Although most authors agree that the pudendal nerve innervates the rhabdosphincter, several anatomical studies have indicated abnormal intrapelvic somatic nerves to the sphincter [8]. Anatomic studies have also shown a partially intrapelvic route for the pudendal nerve branches that go on to innervate the urethral sphincter [7]. The impact of BNS on urinary continence outcomes may be explained by the preservation of these intrapelvic nerves to the rhabdosphincter. As the follow-up time prolongs, the decrease in the difference between BNS and UNS may be attributed to the compensation of other continence mechanisms, such as the pelvic floor musculature, bladder neck sparing, Retzius-sparing RARP and preservation of tissue around the urethra. The most common standard for BNS RP was the presence of low-risk disease [67]. The same but less stringent criteria were used to select patients for UNS: PSA < 10 ng/ml or GS ≤ 6 or normal DRE on the NS side, with or without biopsy core positive information [67]. The decision to perform BNS surgery in these individuals should be made on a case-by-case basis, considering risk stratification based on comprehensive clinical examination, biopsy findings, and imaging results. If BNS is appropriate from an oncologic standpoint, it should be taken into consideration as it might result in better potential for urinary continence following surgery. In addition, UNS can be selected for patients with intermediate- and high-risk diseases who need nerve preservation because its long-term urinary continence outcomes are comparable to those of BNS. Randomized controlled trials comparing BNS with UNS RP are unlikely to be designed for ethical reasons. In the future, to determine the best candidates for BNS RP and UNS RP, prospective multicenter trials with high methodological quality and long-term follow-up for patients with intermediate- and high-risk PCa are required. A deeper understanding of the risk factors for urinary continence may be achievable with sufficiently large sample sizes and multivariate analysis adjusted for specific confounders.

Even though this is the most thorough analysis comparing urinary continence outcomes of BNS to UNS during RP, various limitations must be taken into account when interpreting these data. This meta-analysis is compromised by the absence of randomized controlled trials. Prospective randomized controlled trials, however, are challenging to undertake because the nerve sparing technique may be modified during surgery based on the level of tumor involvement, intraoperative pathology, and other ethical considerations. If the intraoperative frozen pathological results indicate positive surgical margins when the patient undergoes planned BNS, the surgical method may be changed to UNS or NNS for the patient’s ethical consideration. Moreover, our study did not include studies that were not published or in English. Although Egger’s linear regression did not disclose any conclusive evidence of publication bias, assessing publication bias is inherently challenging when there are few studies included. There was significant heterogeneity among studies in terms of urinary continence, which we were unable to fully explain. Heterogeneity can be caused by a variety of factors, including age, prostate volume, membranous urethral length, tumor staging and grading, surgeon experience, and variations in surgical technique. Many studies did not provide enough information to allow for adjustments, and stratification of research outcomes based on surgeon experience, patient age, or other factors was not done. Additionally, variations in the definition of NS status could affect the outcomes. Intrafascial, interfascial, or extrafascial nerve preservation surgery can result in inconsistencies in urinary continence outcomes.

## Conclusion

This meta-analysis demonstrates that BNS results in superior urinary continence outcomes compared to UNS in all selected postoperative follow-up periods. This superiority persists for ≥ 24 mo. We speculate that the preservation of the intrapelvic nerves supplying the rhabdosphincter may be the cause of this relationship. If BNS is deemed appropriate from an oncological perspective, it should be duly considered. High-quality cohort studies are recommended to corroborate the foregoing findings and additional research into the mechanisms of post-RP incontinence.

### Supplementary Information

**Supplementary Material 1.**

**Supplementary Material 2.**

**Supplementary Material 3.**

**Supplementary Material 4.**

**Supplementary Material 5.**

**Supplementary Material 6.**

**Supplementary Material 7.**

**Supplementary Material 8.**

## Data Availability

No datasets were generated or analysed during the current study.
