# Supplementary material for: Is there any difference in urinary continence between bilateral and unilateral nerve sparing during radical prostatectomy? A systematic review and meta-analysis
Source: World J Surg Oncol. 2024 Feb 23;22:66. doi: 10.1186/s12957-024-03340-6 (PMC10885481; doi:10.1186/s12957-024-03340-6)
Supplement: Supplementary file 1 — Supplementary Material 1. [file 12957_2024_3340_MOESM1_ESM.doc]

**Supplementary Figure 1:** Flow diagram of the study

1. Records identified through database searching
2. Total=1249 (PubMed 604, Web of science 645)

398 duplicates excluded

1. 851 records for assessment

528 clearly not relevant

1. 323 full text articles retrieved and reviewed

266 records excluded

171 not relevant

62 no comparison group or clear data

19 reviews, case reports and conferences

14 non-English language

1. 57 studies included in analysis
