# Supplementary material for: Is there any difference in urinary continence between bilateral and unilateral nerve sparing during radical prostatectomy? A systematic review and meta-analysis
Source: World J Surg Oncol. 2024 Feb 23;22:66. doi: 10.1186/s12957-024-03340-6 (PMC10885481; doi:10.1186/s12957-024-03340-6)
Supplement: Supplementary file 2 — Supplementary Material 2. [file 12957_2024_3340_MOESM2_ESM.doc]

**Supplementary Table 1** – Search strategy

| PubMed: from inception to May 31, 2023 | | Web of Science: from inception to May 31, 2023 | |
| --- | --- | --- | --- |
| nerve sparing | 8,585 | nerve sparing | 7,488 |
| prostatectomy | 47,931 | prostatectomy | 54,147 |
| unilateral OR bilateral | 437,236 | unilateral OR bilateral | 386,299 |
| #1 AND #2 AND #3 | 604 | #1 AND #2 AND #3 | 645 |
