# Supplementary material for: Is there any difference in urinary continence between bilateral and unilateral nerve sparing during radical prostatectomy? A systematic review and meta-analysis
Source: World J Surg Oncol. 2024 Feb 23;22:66. doi: 10.1186/s12957-024-03340-6 (PMC10885481; doi:10.1186/s12957-024-03340-6)
Supplement: Supplementary file 3 — Supplementary Material 3. [file 12957_2024_3340_MOESM3_ESM.doc]

**Supplementary Table 2** – Methodology assessment of included studies

| **Prospective** | | | | | | |
| --- | --- | --- | --- | --- | --- | --- |
| Study, year | Baseline continence | Outcome assessment | Comparability of groups | NS assessment | Surgical technique  variations | Other issues |
| Albayrak 2010 [10] | Unclear | Voiding diary, 1-h pad test, following questions: “Do you have a problem with dripping or leaking urine?”; “Over the last 4 weeks how often have you leaked urine?”. Data collected by third party | Data not given to allow  comparison of groups | Not described | No variation described | Selective outcome reporting |
| Asimakopoulos 2019 [11] | All continent | ICS questionnaire；Prospectively collected data； Data collected by third party | Data not given to allow  comparison of groups | Intrafascial or interfascial techniques | Preservation of the bladder neck and puboprostatic ligaments (performed in 50%) | Selective outcome reporting |
| Avulova 2018 [12] | All continent | EPIC-26 questionnaire; Prospectively collected data | BNS vs. UNS/ NNS RARP were younger; Different race, education, cancer related anxiety and D'Amico risk. | NS status recorded prospectively from operative report | No variation described | Unexplained loss to follow-up |
| Berg 2014 [13] | All continent | “Self-reported” continence  according to number of pads; Prospectively collected data | BNS group was younger, with lower biopsy Gleason and clinical stage | Intrafascial or interfascial techniques | No variation described | Unexplained loss to follow-up; Possible selection bias |
| Berry 2009 [14] | No significant difference between groups | UCLA-PCI self-administered;  Prospectively collected data | BNS group was younger, with lower Gleason score and clinical stage, and more likely to perform RARP | NS status recorded into database | No variation described | Unexplained loss to follow-up |
| Bhat 2022 [15] | Unclear | Questions from research staff; Prospectively collected data | Data not given to allow  comparison of groups | Recorded into prospective  database; NS status was graded by the surgeon | Modified posterior rhabdosphincteric reconstruction | Possible selection bias |
| Budaus 2009 [16] | Unclear | ICS, EORTC-QOL-C30 questionnaires; Prospectively collected data | Data not given to allow  comparison of groups | Intrafascial technique | Bladder neck reconstruction (not routine) | Possible selection bias; Unexplained loss to follow-up |
| Burkhard 2006 [17] | Unclear | Patients questioned at follow-up by a third party concerning continence status and pad use | Data not given to allow  comparison of groups | NVB was carefully rolled away from the lateral prostate after incision of the second layer of the endopelvic fascia, that is the periprostatic fascia | Puboprostatic ligament  preservation and bladder neck reconstruction (routine) | Selective outcome reporting |
| Choi 2011 [18] | No significant difference between  groups | EPIC urinary function SF;  Third-party data collection | BNS vs. UNS RARP were younger and more likely to present with clinical stage T1c and Gleason grade 3 + 3 on biopsy | Considered “spared” if at least 70% of NVB remained in situ; Interfascial technique preferred when attempting to spare 100% of NVB | Bladder neck sparing (performed in >50%) | Unexplained loss to follow-up |
| Collette 2021 [19] | Unclear | Prospectively collected data;  EPIC-26 questionnaire | Data not given to allow  comparison of groups | Not described | No variation described | Selective outcome reporting |
| d’Altilia 2022 [20] | All continent | 24h pad test and ICIQ-7 questionnaire were recorded; Third-party data collection | Data not given to allow  comparison of groups | Not described | No variation described | Possible selection bias |
| Dalkin 2006 [21] | Mean urinary function  score 95/100 (whole cohort) | Patient completed UCLA-PCI  SF-36; Third-party data collection | Data not given to allow  comparison of groups | NS status recorded prospectively from operative notes | No variation described | Unexplained loss to follow-up |
| El-Hakim 2015 [22] | Unclear | Prospectively collected data;  Patient-reported pad usage  over 24 h | Data not given to allow  comparison of groups | Not described | Modified posterior reconstruction and bladder neck sparing | Selective outcome reporting |
| Feng 2020 [23] | Unclear | EPIC questionnaire；Prospectively collected data | Data not given to allow  comparison of groups | Not described | Puboprostatic ligament  preservation and bladder neck reconstruction | None |
| Fossati 2017 [24] | Unclear | Prospectively collected data;  Patient-reported pad usage  over 24 h | Data not given to allow  comparison of groups | Not described | No variation described | Selective outcome reporting; Possible effect of trainees performing surgery |
| Geraerts 2013 [25] | Unclear | Prospectively collected data; 3 consecutive days of 0 g urine leak using the 24-h pad test | Data not given to allow  comparison of groups | Not described | No variation described | Unexplained loss to follow-up; Possible selection bias |
| Hatiboglu 2015 [26] | Unclear | Prospectively collected data; No pad usage after catheter removal | Data not given to allow  comparison of groups | Not described | No variation described | Selective outcome reporting |
| Hinata 2014 [27] | Unclear | Prospectively collected data;  Self-administered EPIC  questionnaire | BNS vs UNS RARP were younger with lower PSA level | Intrafascial or interfascial techniques | No variation described | Selective outcome reporting |
| Holze 2019 [28] | All continent | Number of pads used in a 24h  period | Data not given to allow  comparison of groups | Not described | No variation described | Selective outcome reporting |
| Kim 2019 [29] | Unclear | Prospectively collected data; EPIC questionnaire; Data collated by blinded third party | Data not given to allow  comparison of groups | Intrafascial or interfascial techniques | No variation described | Selective outcome reporting |
| Ko 2012 [30] | Unclear | Prospectively collected data;  EPIC questionnaire; Data collated by blinded third party | Data not given to allow  comparison of groups | NS status recorded prospectively into database | Periurethral suspension stitch; Modified posterior reconstruction | Unexplained loss to follow-up; Selective outcome reporting |
| Kohjimoto 2022 [31] | Unclear | EPIC questionnaire; Prospectively collected data | Data not given to allow  comparison of groups | Not described | Periurethral suspension stitch; Posterior wall  reconstruction | Selective outcome reporting |
| Kováčik 2019 [32] | Unclear | Prospectively collected data;  ICIQ-SF questionnaire | Data not given to allow  comparison of groups | Not described | Bladder neck reconstruction; ARVUS (performed in 38%) | None |
| Kowalczyk 2013 [33] | Unclear | Prospectively collected data; EPIC questionnaire; Third-party data collection | Data not given to allow  comparison of groups | Intrafascial, interfascial, or extrafascial techniques | NVB counter-traction and cephalad tension (performed in 44%) | Selective outcome reporting |
| Kung 2015 [34] | All continent | Prospectively collected data; Patient-reported pad usage per day | Data not given to allow  comparison of groups | Not described | No variation described | Possible selection bias |
| Lavigueur-Blouin 2015 [35] | Unclear | Prospectively collected data;  Continence strictly defined as use of a 0-pad scenario | Data not given to allow  comparison of groups | Not described | Posterior rhabdosphincter  reconstruction | Selective outcome reporting |
| Lee 2010 [36] | All continent | “Self-reported” continence according to number of pads; Questions from physician or  research staff | Data not given to allow  comparison of groups | Not described | Posterior rhabdosphincter  reconstruction (performed in 82%) and bladder neck reconstruction (performed in 65%) | Selective outcome reporting |
| Marien 2008 [37] | All continent | UCLA-PCI self-administered; Continence data collected  prospectively | BNS younger | Recorded into prospective  database | No variation described | Selective outcome reporting |
| Nandipati 2007 [38] | Unclear | Self-administered institutional  questionnaire; Prospectively collected data | BNS group were younger | NS status recorded from operative notes | No variation described | Selective outcome reporting |
| Novara 2010 [39] | All continent | Prospectively collected data;  ICIQ-UI SF instrument;  Blinded third party inserted  score into database | Data not given to allow  comparison of groups | Intrafascial technique  (“in most patients”) | Preservation of bladder neck and puboprostatic ligaments (routine) | Selective outcome reporting |
| Nyarangi-Dix 2020 [40] | Unclear | Continence data collected  prospectively | Data not given to allow  comparison of groups | Not described | Preservation of bladder neck | Selective outcome reporting |
| Pagliarulo 2020 [41] | Unclear | Continence was defined as no need for any pad, at any time | Data not given to allow  comparison of groups | Intrafascial or interfascial techniques | No variation described | Possible selection bias |
| Pick 2011 [42] | No significant difference in baseline AUA-SS | Prospectively collected data;  selected questions from EPIC-  26 and AUA-SS | BNS group was younger, with lower PSA level, and more likely to be cT1 | Antegrade interfascial  approach | No reconstructive measures used | Unexplained loss to follow-up; Selective outcome reporting |
| Reichert 2022 [43] | 25% incontinent  preoperatively (whole cohort) | Prospectively collected data; ICS questionnaire; Data collated by blinded third party | Data not given to allow  comparison of groups | Not described | No variation described | Selective outcome reporting |
| Rigatti 2012 [44] | Unclear | Prospectively collected data; ICI-Q | Data not given to allow  comparison of groups | Not described | No variation described | Selective outcome reporting |
| Sammon 2013 [45] | Unclear | Data collected prospectively; Self-administered questionnaire according to number of pads | Data not given to allow  comparison of groups | Standard, intrafascial or interfascial techniques | Posterior reconstruction  (performed in 80%) and  bladder neck reconstruction (performed in 7%) | Selective outcome reporting |
| Scarcia 2018 [46] | Unclear | Data collected prospectively; Continence defined as use of 0-1 pad per day | Data not given to allow  comparison of groups | Bilateral intrafascial and unilateral interfascial technique | Posterior reconstruction | Selective outcome reporting |
| Steineck 2015 [47] | 5% incontinent  preoperatively (whole cohort) | Patients questioned at follow-up by a third party concerning continence status and pad use | Data not given to allow  comparison of groups | Intrafascial or interfascial techniques | Bladder neck sparing (performed in >79%) | Selective outcome reporting |
| Suardi 2012 [48] | Unclear | Patient-reported pad usage over 24h; Prospectively collected data | Data not given to allow  comparison of groups | Not described | No variation described | Selective outcome reporting |
| Talcott 1997 [49] | Preoperative incontinence reported as “low” | Patient-reported pad usage  over 24 h | BNS group was younger, with lower PSA | Retrospective analysis of  operative notes to establish NS status | No variation described | Unexplained loss to follow-up |
| Theissen 2019 [50] | Unclear | Pad-test; Questions from physician or research staff | Data not given to allow  comparison of groups | Not described | No variation described | Selective outcome reporting |
| Toren 2009 [51] | No significant difference in baseline continence score | Continence data collected prospectively; PORPUS questionnaire | BNS group: Lower Gleason | Established retrospectively from operation report | No variation described | Selective outcome reporting |
| Tsikis 2017 [52] | All continent | UCLA-PCI questionnaire; Prospectively collected data | Data not given to allow  comparison of groups | NS status recorded prospectively into database | No variation described | Selective outcome reporting |
| Tzou 2009 [53] | No significant preoperative incontinence | UCLA-PCI patient completed survey; Sent to third party for data collection | Data not given to allow  comparison of groups | NS status recorded prospectively into database | No variation described | Unexplained loss to follow-up |
| Van der Poel 2009 [54] | 9% incontinent  preoperatively | EORTC QLQ-C30 and Prostate Cancer Module PR25  questionnaires | Data not given to allow  comparison of groups | Not described | Fascial preservation | None |
| Van der Slot 2023 [55] | All continent | ICIQ-UI SF questionnaire; EPIC-26; Prospectively collected data; | Data not given to allow  comparison of groups | Not described | No variation described | Unexplained loss to follow-up |
| **Retrospective** | | | | | | |
| Chung 2020 [56] | Unclear | “Self-reported” continence according to number of pads; Data collated by blinded surgeon | Data not given to allow  comparison of groups | Not described | Rocco stitch and anterior reconstruction (67%) | Selective outcome reporting |
| Fosså 2019 [57] | No significant difference between  groups | EPIC-26 questionnaire; Details of questionnaire delivery not provided | Data not given to allow  comparison of groups | Intrafascial or interfascial techniques | No variation described | Unexplained loss to follow-up |
| Greco 2011 [58] | Unclear | ICIQ-UI SF self-administered  questionnaire | BNS vs UNS LRP: more operation time, mean blood loss and transfusion; age/PSA/ Gleason no significant difference | Intrafascial technique | Puboprostatic ligament  preservation | None |
| Hinata 2019 [59] | Unclear | EPIC questionnaire | Data not given to allow  comparison of groups | Intrafascial or interfascial techniques | HA/CMC application (performed in 5%) | Selective outcome reporting |
| Kadono 2015 [60] | Unclear | ICIQ-SF questionnaire; Self-administered 24-hr pad tests | BNS vs UNS RARP were lower D'Amico risk | Intrafascial or interfascial techniques | Double-layered posterior reconstruction | Unexplained loss to follow-up; Selective outcome reporting |
| Lee 2013 [61] | Unclear | “Self-reported” continence according to number of pads | Data not given to allow  comparison of groups | Extrafascial (90%) or intrafascial techniques (10%) | No variation described | Selective outcome reporting |
| Noël 2022 [62] | Unclear | Continence was defined as the absence of uncontrolled leakage of urine | Data not given to allow  comparison of groups | Not described | dHACM (performed in all) | Possible selection bias |
| Palisaar 2015 [63] | Unclear | Defined ≤ 1 pad/day in 24 h as continent | Data not given to allow  comparison of groups | Intrafascial technique | Sparing of bladder neck and puboprostatic ligaments (routine) | Selective outcome reporting |
| Punnen 2014 [64] | All continent | “Self-reported” continence according to number of pads | Data not given to allow  comparison of groups | Not described | Retropubic urethral sling placement (66%) | Selective outcome reporting |
| Shikanov 2011 [65] | Unclear | UCLA-PCI self-administered validated questionnaire. | Data not given to allow  comparison of groups | Interfascial or extrafascial techniques | No variation described | Selective outcome reporting |
| Wang 2014 [66] | Unclear | Patient-reported pad usage  over 24 h | Data not given to allow  comparison of groups | Intrafascial technique | No variation described | Selective outcome reporting |
| EPIC-26 = Expanded Prostate Cancer Index Composite questionnaire; BNS = bilateral nerve sparing; UNS = unilateral nerve sparing; NNS = non-nerve sparing; NS = nerve sparing; UCLA-PCI = University of California, Los Angeles, Prostate Cancer Index; RARP = robot-assisted radical prostatectomy; ICS = International Continence Society; ICIQ-UI SF = International Consultation of Incontinence Questionnaire-Urinary Incontinence Short Form; EORTC QLQ-C30 = European Organisation for Research and Treatment of Cancer Quality of Life Questionnaire; NVB = neurovascular bundle; EPIC-26 = Expanded Prostate Cancer Index Composite questionnaire; ARVUS = Advanced Reconstruction of Vesicourethral Support; AUA-SS = American Urological Association Symptom Score; PSA = prostate-specific antigen; ICI-Q = International Consultation on Incontinence-questionnaire; PORPUS = Patient Oriented Prostate Utility Score; EORTC QLQ-PR25 = European Organisation for Research and Treatment of Cancer Quality of Life Questionnaire-Prostate Module; LRP = laparoscopic radical prostatectomy; HA/CMC = hyaluronic acid-carboxymethyl cellulose membrane; dHACM = dehydrated human amnion/chorion membrane; | | | | | | |
