# Supplementary material for: Is there any difference in urinary continence between bilateral and unilateral nerve sparing during radical prostatectomy? A systematic review and meta-analysis
Source: World J Surg Oncol. 2024 Feb 23;22:66. doi: 10.1186/s12957-024-03340-6 (PMC10885481; doi:10.1186/s12957-024-03340-6)

**Supplementary Figure 3** – Sensitivity analysis: forest plot of continence rates for bilateral nerve sparing (BNS) versus unilateral nerve sparing (UNS) at 12 mo (**a**) and ≥ 24 mo (**b**) in prospective studies only.


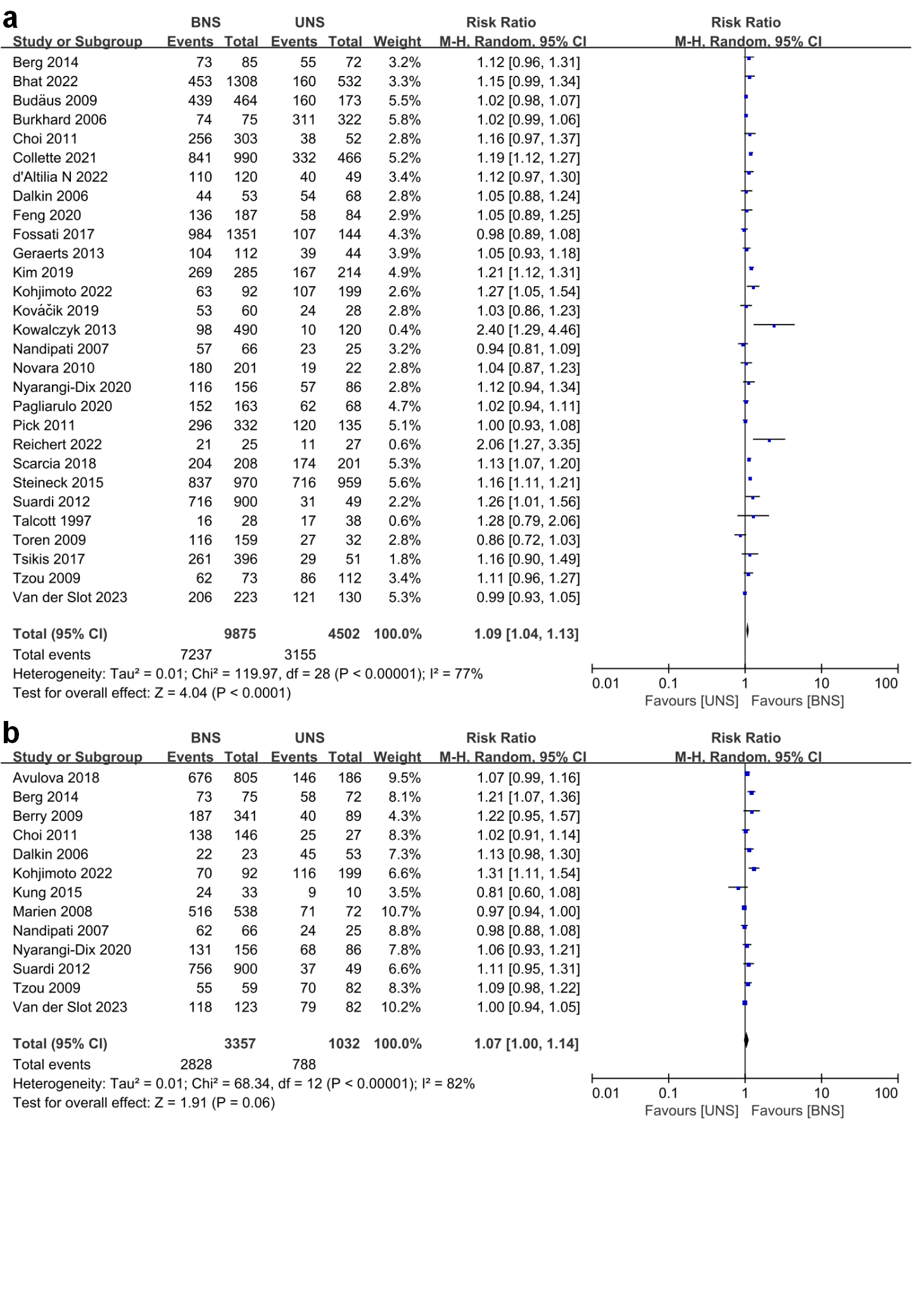

Supplement: Supplementary file 5 — Supplementary Material 5. [file 12957_2024_3340_MOESM5_ESM.doc]
