# Supplementary material for: Is there any difference in urinary continence between bilateral and unilateral nerve sparing during radical prostatectomy? A systematic review and meta-analysis
Source: World J Surg Oncol. 2024 Feb 23;22:66. doi: 10.1186/s12957-024-03340-6 (PMC10885481; doi:10.1186/s12957-024-03340-6)

**Supplementary Figure 6:** Statistical evaluation of funnel plot asymmetry with Egger linear regression for bilateral nerve sparing (BNS) versus unilateral nerve sparing (UNS) at ≤ 1.5 mo (**a**), 3-4 mo (**b**), 6 mo (**c**), 12 mo (**d**) and ≥ 24 mo (**e**).


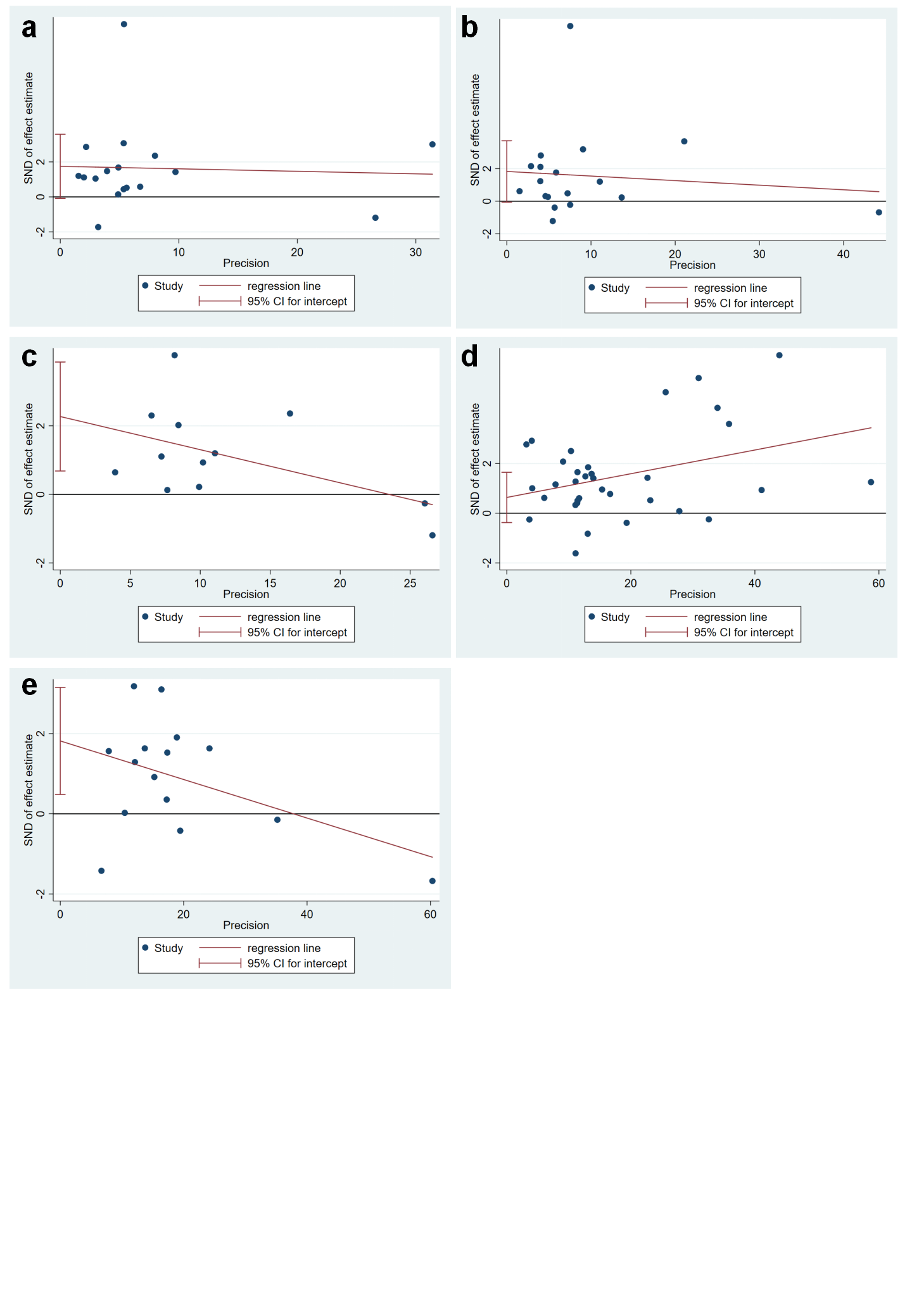

Supplement: Supplementary file 8 — Supplementary Material 8. [file 12957_2024_3340_MOESM8_ESM.doc]
